# Role of p73 in Alzheimer disease: lack of association in mouse models or in human cohorts

**DOI:** 10.1186/1750-1326-8-10

**Published:** 2013-02-15

**Authors:** Badri Vardarajan, David Vergote, Fadel Tissir, Mark Logue, Jing Yang, Nathalie Daude, Kunie Ando, Ekaterina Rogaeva, Joseph Lee, Rong Cheng, Jean-Pierre Brion, Mahdi Ghani, Beipei Shi, Clinton T Baldwin, Satyabrata Kar, Richard Mayeux, Paul Fraser, André M Goffinet, Peter St George-Hyslop, Lindsay A Farrer, David Westaway

**Affiliations:** 1 Department of Medicine (Biomedical Genetics), Boston University School of Medicine, 715 Albany Street, Boston, MA 02118, USA; 2 Centre for Prions and Protein Folding Diseases, University of Alberta, Edmonton, AB T6G 2M8, Canada; 3 Developmental Neurobiology, Institute of Neuroscience, Université catholique de Louvain, B1200, Brussels, Belgium; 4 Laboratory of Histology, Neuroanatomy and Neuropathology, Université Libre de Bruxelles, B1070, Brussels, Belgium; 5 Centre for Research in Neurodegenerative Diseases, Departments of Medicine, Laboratory Medicine and Pathobiology, Medical Biophysics, University of Toronto, and Toronto Western Hospital Research Institute, Toronto, ON, Canada; 6 The Taub Institute on Alzheimer's Disease and the Aging Brain, The Gertrude H. Sergievsky Center, College of Physicians Surgeons, Department of Epidemiology, Mailman School of Public Health, Columbia University, New York, NY, USA; 7 Center for Human Genetics, Boston University School of Medicine, Boston, MA, USA; 8 Cambridge Institute for Medical Research and Department of Clinical Neurosciences, University of Cambridge, Cambridge, UK; 9 Departments of Neurology, Ophthalmology, Genetics & Genomics, Epidemiology, and Biostatistics, Boston University Schools of Medicine and Public Health, Boston, MA, USA

**Keywords:** P73, Alzheimer’s disease, Animal models, GWAS

## Abstract

**Background:**

P73 belongs to the p53 family of cell survival regulators with the corresponding locus *Trp73* producing the N-terminally distinct isoforms, TAp73 and DeltaNp73. Recently, two studies have implicated the murine *Trp73* in the modulation in phospho-tau accumulation in aged wild type mice and in young mice modeling Alzheimer’s disease (AD) suggesting that *Trp73*, particularly the DeltaNp73 isoform, links the accumulation of amyloid peptides to the creation of neurofibrillary tangles (NFTs). Here, we reevaluated tau pathologies in the same TgCRND8 mouse model as the previous studies.

**Results:**

Despite the use of the same animal models, our *in vivo* studies failed to demonstrate biochemical or histological evidence for misprocessing of tau in young compound Trp73^+/-^ + TgCRND8 mice or in aged Trp73^+/-^ mice analyzed at the ages reported previously, or older. Secondly, we analyzed an additional mouse model where the DeltaNp73 was specifically deleted and confirmed a lack of impact of the DeltaNp73 allele, either in heterozygous or homozygous form, upon tau pathology in aged mice. Lastly, we also examined human *TP73* for single nucleotide polymorphisms (SNPs) and/or copy number variants in a meta-analysis of 10 AD genome-wide association datasets. No SNPs reached significance after correction for multiple testing and no duplications/deletions in *TP73* were found in 549 cases of AD and 544 non-demented controls.

**Conclusion:**

Our results fail to support P73 as a contributor to AD pathogenesis.

## Background

Neuropathological staging of Alzheimer’s Disease (AD) tissue indicates a preeminent role for sequential tau deposition, and tau pathology is well correlated with dementia scores [1-3]. On the other hand, genetic studies indicate the primacy of changes in Aβ synthesis as a key causal factor that is often described as being “upstream” of tau. Because of the exponential incidence of AD in the industrialized countries, defining links between Aβ and tau would be of great importance, from both theoretical and practical points of view. To this end, introduction of mutations affecting APP processing into transgenic animal models can induce florid amyloid pathology and modest neuritic tau pathology only in the immediate vicinity of plaques [4-7], but is not associated with the development of florid neurofibrillary tangles (NFTs) as characteristically seen in AD. This inability of APP transgenic mice to display florid tau pathologies at sites distal to Aβ assemblies is a disadvantage in contexts where candidate therapeutics are being tested and has led to increased interest in double- and triple-transgenic models of human neuropathologies [8-10]. Co-existing and potentially synergistic Aβ and tau pathologies can be obtained by simultaneously introducing separate human APP and tau transgenes (albeit often using a mutant form of human tau), or by delivering Aβ into the central nervous system of TgTau mice [8,11-13]. While the potential of mutant and wild type (wt) human tau to form NFTs *in vivo* is well established [14-16], progress on attaining an analogous situation for endogenous murine tau protein has proven more challenging. The origin of this attenuated pathogenic potential of the murine protein is, however, not clear as both recombinant human and mouse proteins are able to form tangle-like structures *in vitro*[17] and, *in vivo,* hyperphosphorylated mouse tau accumulates in dystrophic neurites adjacent to amyloid plaques such as, in sections from TgCRND8 mice [4]. Besides “3R” and “4R” tau mRNA splicing variants (encoding different numbers of microtubule binding domains) under developmental and neuroanatomical control ([18] and references therein), one possibility for the failure of wt mouse tau to form NFTs is a distinction between mice and humans within a chemical pathway that acts to link Aβ accumulation to tau. In this context, the finding that mice haploinsufficient for the murine *Trp73* locus, a member of the *Trp53* gene family enriched in the nervous system, are prone to develop tau pathology in presence of an APP transgene [19] attracted a degree of attention [20]. In addition to the importance of this biological question, the young age of the 1.5 to 2-month old animals reported to have abnormal tau species derived from the wt murine tau expressed at endogenous level was notable [19]. Plaque onset within 100% of animals is typically at 3 months of age in TgCRND8 mice [4] and 1.5-2-month time-points are also earlier than that reported for onset of pathology in several transgenic mouse lines overexpressing germline mutant forms of tau [11,16,21]. In consequence, we were drawn to reevaluate tau pathologies reported in *Trp73*^*+/-*^/TgCRND8 compound transgenic mice [19,22]. For this purpose we used a series of tau-based protein and histochemical assays in compound transgenic and control mice of the same origin of the same age or older than those used in the prior report. Our studies failed to detect convincing pathologies in cohorts of compound transgenic mice. Also, because other studies found borderline evidence for an association between genetic or copy number variants in the human p73 gene (TP73) and risk for AD [23,24], we evaluated the possible association of single nucleotide polymorphisms (SNPs) and copy number variants (CNVs) in the human *TP73* gene with AD in several large datasets. The results presented here fail to define a genetic association between the p73 locus and AD pathology in mouse models and in human cohorts.

## Results

### Studies in *Trp73*^+/-^ + TgCRND8 compound mutant mice

**Figure 1:**
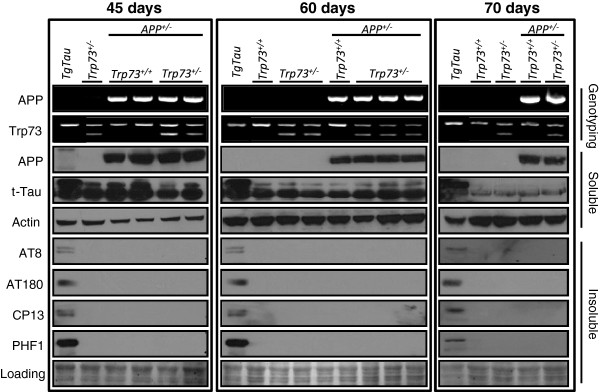
**Biochemical analysis of tau species in control and compound mutant young mice. **The three columns display protein samples derived from litters of increasing ages (45, 60 and 70 days old). The human APP695 transgene (“HuAPP”) and Trp73 genotypes were confirmed by PCR-based methodologies (top two rows) as described in material and methods. Western blot analysis is shown for transgene-encoded human APP (row 3: 6E10 antibody), with total tau (row 4) and actin (row 5) immunoblots serving as a loading control in soluble fractions. Tau antibodies, AT8, AT180, CP13 and PHF-1 (rows 6-9, respectively), were used to evaluate pathological forms of tau in each sample in insoluble fractions. A tau transgenic sample (left-hand lane) was included in the immunoblot series for each time-point as a positive control (TgTau^P301L ^mouse, age 540 days). “+/-“ in the case of *Trp73 *and TgAPP indicates that these mice were heterozygous for null allele and hemizygous the TgCRND8 APP transgene array, respectively. Loading controls (“Loading”; row 10) consist of images of Ponceau red-stained membranes taken immediately after blotting.

To assess and/or replicate the results obtained previously [19,22], we obtained mice bearing the same null allele of murine *Trp73* used in the original report, and intercrossed these animals with the TgCRND8 line of APP transgenic mice. We note here that high levels of expression of APP695 are associated with increased mortality in the parental TgCRND8 mice, as is the case for other TgAPP mice. This effect is not necessarily dependent upon the inclusion of familial AD mutations within APP that predispose to amyloid formation, but can be influenced by inbred strain background [4,25-27]. In our studies the resultant C3H/C57BL6 hybrid genetic background in the compound mice is similar to backgrounds used previously to study TgCRND8 mice [4]. Compound *Trp73*^+/-^ + TgCRND8 animals generated by our crosses expressed the APP transgene appropriately (compared to TgCRND8; Figure 1 rows 1 and 3) and, at weaning, 15 compound mice were observed (versus 12 expected) out of a total of 49 mice. The rate of occurrence of the postnatal mortality effect in the *Trp 73*^+/-^ + TgCRND8 compound mutant mice (4 events – d35, d44, d49, d65 - out of 15 animals genotyped) was not distinguishable statistically from TgCRND8 mice with a homozygous wild type (wt) *Trp73* genotype (3 events - d30, d53, and d73 - out of 10 genotyped TgCRND8 neonates), assessed here up to postnatal day 110.

*Trp73*^+/-^ + TgCRND8 compound mutant mice, along with age-matched control *Trp73*^+/-^, TgCRND8 and wild type littermates, were then examined for tau alterations. Cohorts of adolescent mice were either of the same age (45-60 days) or older (70 days) than mice described by Wetzel *et al*[19]. Four well-characterized antibodies detecting phospho-specific epitopes of the endogenous mouse tau protein were used for analyses of biochemical and immunohistochemical alterations in the compound mice (AT8, CP13, AT180 and PHF1). MC1, a conformation-specific antibody, was also used in some immunohistochemical analyses. As a positive control for the analyses of pathological processing and deposition of murine tau in the compound *Trp73*^+/-^ + TgCRND8 mice, we used mice expressing either P301L or G272V/P301S mutant tau transgenes (designated TgTau^P301L^ and Tg30tau, respectively), both of which develop extensively characterized neuronal tau pathologies [28-30] (Figure 1, row 3). In the former case the tau accumulation extends to glia and oligodendrocytes, providing a broad point of reference for tau pathologies.

**Figure 2:**
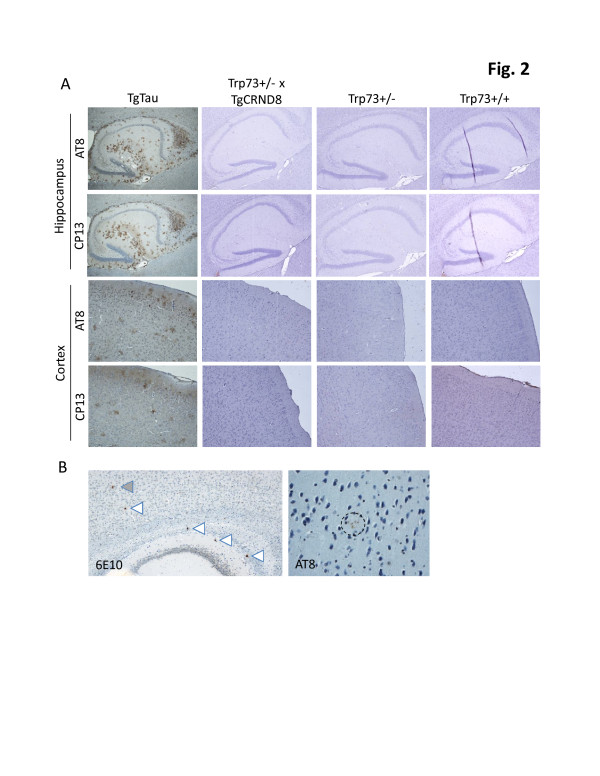
**Immunohistochemical analysis of tau species in control and compound mutant young mice. A**: Tau pathology was assessed histologically in the hippocampal formation (top; 4 x objective) and cortex (bottom; 10 x objective). The left-hand column shows a control tau transgenic mouse (TgTau^P301L^) with tau-positive astrocytic plaque and neurofibrillary tangle morphologies. Immunodetected structures are denoted by brown (DAB) staining, in accord with previous analyses of mice of this age (540 days) [28]. No staining is apparent in compound mutant mice aged 60 days (2^nd^ column) or in *Trp73*+/- or wild type aged matched littermate controls (3^rd ^and 4^th ^columns, respectively) for AT8 and CP13 phospho-tau specific antibodies. **B**: Detection of neuritic pathology. Left hand field (4x) represents the area of hippocampus and cortex from a sagittal section of 70 day-old compound transgenic mouse bearing a total of 5 amyloid plaques (arrows) visualized with 6E10 antibody. Right hand panel (40x) shows one of the cortical plaques (grey arrow, left panel) that has developed neuritic tau pathology. The plaque vicinity is shown (circle with a dashed line) and the neuritic pathology is manifest by dot-like immunostaining within the plaque (see also Additional file 2: Figure S2).

Biochemical analysis of our compound *Trp73*^+/-^ + TgCRND8 mice (45-70 day-old) failed to demonstrate significant levels of insoluble tau species with four phospho-specific anti-tau antibodies (Figure 1; Additional file 1: Figure S1 for a longer autoradiographic exposure), whereas insoluble tau species were detected in the TgTau^P301L^ control mice. Tau immunostaining of hippocampus and frontal cortex (the two main brain regions displaying pathology in AD brains and animal models) failed to reveal any staining in paraffin-embedded, fixed tissue from compound *Trp73*^+/-^ + TgCRND8 mice, either at (45-60 days old) or after (70 days old) the ages reported by Wetzel *et al.* (Figure 2A and Additional file 2: Figure S2). These analyses were performed with AT8, CP13 MC1 and AT180 antibodies. As anticipated, novel tau pathologies were absent in negative control *Trp*73^+/-^ and *Trp*73^+/+^ littermates (Figure 2A and Additional file 2: Figure S2), as was the case for TgCRND8^+/-^ littermates, in accord with previous analyses [4] (Additional file 2: Figure S2 and data not shown). In contrast, florid tau pathologies were detected in sections from TgTau^P301L^ control animals processed in parallel. As a further control we assessed amyloid plaques and any associated dystrophic changes. As anticipated from our earlier studies, the time period 60-90 days is on the cusp for detection of plaques in TgCRND8 mice, and we were unable to detect amyloid deposits in 60 day-old compound transgenic animals (data not shown). Sparse amyloid deposits were present in 70 day-old compound transgenic animals (Figure 2B), as was the case for TgCRND8 mice, and the analysis of these 70 day-old animals verified our ability to detect tau immunostaining in the form of dystrophic neurites in animals bearing the TgCRND8 transgene array (Figure 2B), demonstrating that the genetic background used for these experiments is not refractory *per se* to the development of tau pathologies.

**Figure 3:**
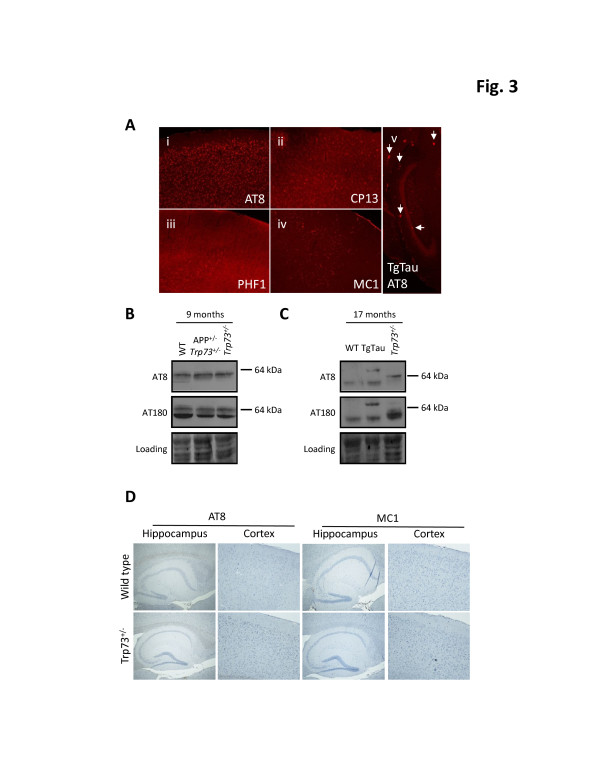
**Biochemical and histochemical analysis of tau species in older compound and *Trp73 *mutant mice. A**: Fluorescent immunostaining of a 85 day-old compound Tg *Trp73*^+/- ^plus TgAPP^+/- ^mouse hemizygous for the TgCRND8 transgene array (i-iv) with tau antibodies AT8, CP13, PHF1 and MC1. Decreasing amounts of somatodendritic staining are present with AT8, CP13 and PHF1 antibodies, respectively. Thread-like staining is not seen. Staining with MC1 (iv) may correspond to detection of blood vessels and a similar pattern was apparent with an anti-Aβ antibody 6E10 applied to a TgTau^P301L^ mouse lacking an APP transgene (not shown). Panel v shows a section, processed in parallel, from a TgTau^P301L ^mouse. Note that the panels i-iv were overexposed compared to panel v to demonstrate intensity differences between pathological features and neuroanatomical structures: a tangle bearing neuron is shown in the granular layer of the dentate gyrus (horizontal arrow) and glial plaques in the subiculum and molecular layer (vertical arrows). **B**: Western blot analyses of phospho-specific tau species in insoluble protein fractions derived from animals at 270 days of age. Tau antibodies, AT8 and AT180, (rows 1-2, respectively), were used. **C**: Insoluble tau fractions of brain homogenates prepared from a *Trp73*^+/- ^mouse at age 17 months were examined by western blotting with antibodies as labeled. **D**: Histochemical analyses with AT8 and CP13 antibodies of a *Trp73*^+/- ^mouse at age 17 months did not reveal tau immunostaining. Positive controls are as per Figure 2A. An age-matched wt mouse is also shown for these analyses of paraffin-embedded tissue. 4 x and 10 x objective was used for hippocampus and cortex, respectively. Loading controls in **B **and **C **were as per Figure 1.

In addition to paraffin-embedded tissue we also performed immunostaining on frozen brain sections from 85 day-old compound mice using three phospho-specific tau antibodies (AT8, CP13 and PHF1) and with one conformation-specific antibody (MC1). Our results were negative with MC1 and PHF1 antibodies, while AT8 and CP13 antibodies (which have overlapping epitopes) yielded some staining of cortical neurons (Figure 3A). However, this staining differed from the focal tau pathologies evident in TgTau^P301L^ control mice (Figure 3A, panel v).

To assess whether our mouse cohorts had a slowed disease progression, additional analyses were performed on animals approximately four times older than the mice described by Wetzel *et al.* Here immunoblots show that 9 month-old *Trp*73^+/-^ mice have no signal differences in detergent insoluble fractions probed with AT8 and AT180 antibodies (Figure 3B) compared to wild type littermates. Presence of an APP transgene in a *Trp73*^+/-^ mouse (*i.e.* a compound mouse of the same genotype as analyzed in Figure 1) did not have an obvious impact on the properties of these tau species. Interestingly, the material in the soluble tau fractions (Additional file 3: Figure S3) showed minor species of slower electrophoretic mobility in *Trp73*^+/-^ animals compared to wild type littermates, as detected with AT8 and CP13 antibodies. Similar species were evident in the compound mouse of the same age. No such distinction from wt controls was detected with PHF1 or AT180 antibodies.

**Figure 4:**
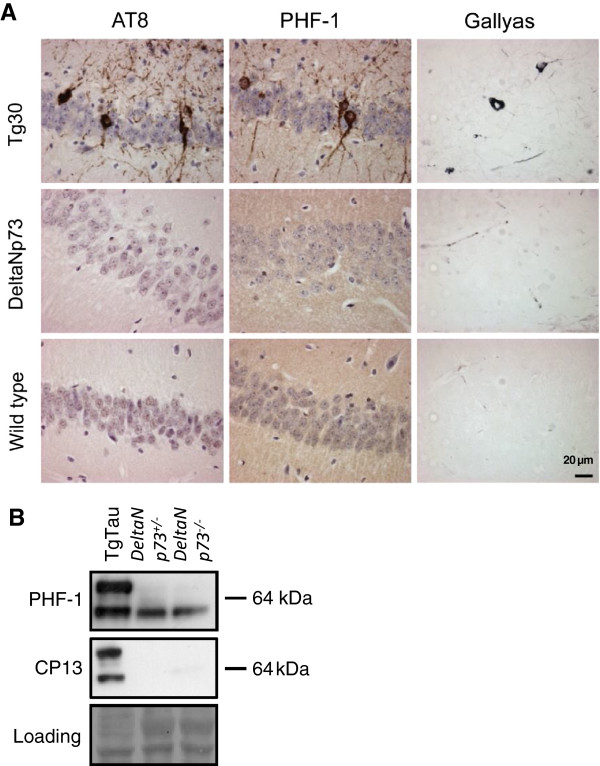
**Analysis of tau species in aged delta NP73 null mice. A**: Histochemical analysis of the hippocampus of 17 month-old DeltaNp73 homozygous mutant mouse (middle row) and a wildtype littermate control (bottom row). Immunohistochemitry with AT8 and PHF-1 antibodies (left and middle columns, respectively) and Gallyas staining (right column) were used to examine tau pathology in these mice. Tau transgenic Tg30 mice were used as a positive control for staining (top row) with Gallyas-positive aggregates and a somatodendritic accumulation of phosphotau being observed in TgTau but not in DeltaNp73 mice. **B**: Immunoblot analysis of 17 month-old DeltaNp73 hemizygous and homozygous mutant mice using phosphotau specific antibodies. Tau transgenic Tg30 mice were used as a positive control (left lane). Loading controls (bottom row) consist of images of Ponceau red-stained membranes taken immediately after blotting.

A direct effect of Trp73 hemizygosity on tau pathology was also reported in aged mice (16-18 months), independent of the presence of an APP transgene [19,22]. This phenotype was attributed to lack of DeltaNp73, the sole p73 isoform present within the central nervous system [31]. To investigate this observation, we first carried out RT-PCR experiments to assess that the DeltaNp73 transcripts deriving from the promoter in intron 3 of the Trp73 locus accounted for all p73 mRNAs expressed in the aged mouse brain; this proved to be the case (data not shown). Next, we examined *Trp*73^+/-^ mice from our own cohorts at similar ages, and subsequently we analyzed aged mice with a selective knockout of the DeltaNp73 isoform [32]. In 17 month-old *Trp*73^+/-^ mice, a minor fraction of tau immunoreactivity detected with AT8 and AT180 antibodies in insoluble fractions was qualitatively different in the *Trp*73^+/-^ mouse compared with control samples from TgTau mice encoding the 2N, 4R isoform of human tau (Figure 3C), but by standard histological analysis of paraffin-embedded fixed tissue we were unable to detect any abnormal tau structures in the *Trp*73^+/-^ mice. Here we used antibodies recognizing abnormally phosphorylated tau species (Figure 3D). In a DeltaNp73-specific knockout mouse model where both heterozygous and homozygous DeltaNp73 deficient mice survive to adulthood [32], brains were examined from old (15-24 months) DeltaNp73 heterozygous mice and from wild type littermates. Of importance, our analyses also included DeltaNp73 homozygotes, hence going beyond the hemizygous state reported in prior analyses [19]. Nonetheless, immunohistochemical signal in mutant samples did not differ significantly from that in control samples, whereas strong somatodendritic phosphotau immunoreactivity and Gallyas positive tangles were evident in neurons of tau (Tg30tau) control transgenic mice analyzed in parallel (Figure 4A). Moreover, we were not able to detect any accumulation of insoluble hyperphosphorylated tau by immunoblotting in homozygous or hemizygous DeltaNp73-deficient animals (Figure 4B).

A further experimental variable between our study and the previous reports concerns the mode of euthanasia. In the study presented to this point, mice were euthanized by cervical dislocation while Wetzel *et al*. and Cancino *et al*. used anaesthetic (sodium pentobarbital) overdose. Previous studies by other groups have shown that anaesthesia can induce tau immunoreactivity for antibodies AT8, AT180, and CP13 even in the absence of human tau or APP transgenes [33]. In experiments here we examined the tau pathology in 45 day-old adolescent wt mice (C3H/C57BL6 background) euthanized either by cervical dislocation or sodium pentobarbital administration. Our results showed an increased phosphorylation of tau in detergent insoluble extracts from sodium pentobarbital-treated animals versus animals euthanized by cervical dislocation (as used for all our studies prior to this point) (Additional file 4: Figure S4). Thus an effect of euthanasia by anesthetic overdose can generate phospho-tau species (encompassing Ser 202 and 396/404 as detected by CP13 and PHF1 antibodies) at the time of tissue harvest.

### Assessing genetic association analyses in human populations

To examine the contribution of the P73 locus in the development of AD in humans, we tested whether there was meaningful support for 54 genotyped or reliably imputed SNPs in *TP73* to be a risk factor for AD. This analysis used 10 genome wide association studies (GWAS) datasets (listed in Table 1) that constituted the discovery sample in a previously published GWAS [34]. Inspection of results within individual datasets revealed 28/540 (5.2%) of the comparisons were nominally significant (see Additional file 5: Table S1) which is almost exactly the number of results expected to attain this level of significance due to chance alone. More than one-half of the significant findings were observed in one of the smallest datasets (ADNI). None of the SNPs were nominally significant in the meta-analysis (Table 2). In addition, there was no evidence for association on a gene-based level using the VEGAS method (P>0.95). Finally, no deletions were discovered among 549 AD cases and 544 non-demented controls as a cause of the potential haploinsufficiency of the *TP73* locus as inferred by Wetzel *et al*[19]. These CNV experiments also did not identify any duplication events encompassing *TP73*.

**Table 1:** Subject characteristics

**Cohort**	**AD cases**			**Controls (N)**		
	**N**	**Age at onset**	***APOE *ε2/ε3/ε4**	**N**	**Age at exam**	***APOE *ε2/ε3/ε4**
		**(mean ± SD)**	**(allele %)**		**(mean ± SD)**	**(allele %)**
ACT	566	83.90 (4.8)	0.05 / 0.69 / 0.26	1696	81.08 (6.0)	0.08 / 0.81 /0.11
ADC1	1566	72.47 (7.1)	0.03 / 0.55 / 0.42	515	75.00 (8.0)	0.08 / 0.76 / 0.16
ADC2	738	73.19 (7.1)	0.03 / 0.57 / 0.39	160	75.68 (7.9)	0.09 / 0.75 / 0.16
ADNI	268	75.30 (7.2)	0.03 / 0.55 / 0.42	173	78.6 (5.5)	0.08 / 0.79 / 0.14
GenADA	669	74.59 (6.2)	0.04 / 0.58 / 0.38	713	74.21 (7.0)	0.08 / 0.79 / 0.13
MIAMI	1186	74.06 (7.8)	0.03 / 0.61 / 0.36	1135	74.00 (8.3)	0.08 / 0.80 / 0.12
MIRAGE	509	71.16 (6.5)	0.04 / 0.60 / 0.36	753	72.04 (7.2)	0.06 / 0.72 / 0.23
NIA-LOAD	1811	73.57 (6.7)	0.02 / 0.51 / 0.46	1575	73.99 (8.5)	0.07 / 0.73 / 0.20
OHSU	132	86.10 (5.5)	0.07 / 0.70 / 0.23	153	83.86 (7.6)	0.10 / 0.82 / 0.08
TGEN	864	74.91 (7.2)	0.04 / 0.57 / 0.40	493	80.19 (8.7)	0.10 / 0.79 / 0.11
**TOTAL**	**8309**	**--**	**--**	**7366**	**--**	**--**

**Table 2:** **Association of AD with *TP73 *SNPs in 10 GWAS datasets**

**SNP**	**Position**	Alleles *	**RAF**	**Meta P**	**Direction**
rs2821021	3,524,142	A/G	0.70	0.71	+--+-+-+-+
rs4648538	3,543,492	T/C	0.27	0.56	--+-+-+-++
rs4276857	3,544,345	T/C	0.73	0.57	++-+-+-+--
rs1128474	3,545,611	G/A	0.78	0.22	-+-+---+--
rs1885864	3,554,987	T/C	0.19	0.24	+-+++++-++
rs3818330	3,555,427	T/G	0.19	0.24	+-+++++-++
rs12027041	3,591,447	C/G	0.47	0.98	+-++-----+
rs3765770	3,635,980	A/G	0.036	0.76	-+++--++--
rs747828	3,636,225	T/C	0.96	0.78	+---++--++
rs747827	3,636,346	A/C	0.96	0.79	+---++--++
rs2236365	3,644,857	C/G	0.056	0.77	--++---++-
rs6664760	3,648,267	T/C	0.96	0.79	+---++--++
rs6695978	3,648,344	A/G	0.041	0.80	-+++--++--
rs9662633	3,649,561	A/G	0.057	0.64	-+++--++--
rs12562437	3,651,030	T/C	0.041	0.80	-+++--++--
rs10910018	3,651,408	A/G	0.042	0.83	-+++-+-+--
rs12117836	3,657,758	A/G	0.37	0.91	--+++--++-
rs2298222	3,659,656	A/G	0.30	0.80	---++--+++
rs3737589	3,662,844	A/G	0.63	0.88	++---++--+
rs12120656	3,665,866	T/G	0.37	0.89	--+++--++-
rs4648554	3,668,003	T/C	0.63	0.91	++---++--+
rs12128253	3,668,752	A/C	0.75	0.77	-++--++---
rs10797410	3,669,200	A/G	0.63	0.92	++---++--+
rs10910022	3,669,499	A/G	0.97	0.74	+---++--++
rs1181889	3,671,026	T/C	0.88	0.95	+--++-+--+
rs1181888	3,671,790	A/G	0.88	0.93	+--++-+--+
rs12128669	3,676,076	T/C	0.25	0.78	---++--+++
rs1181885	3,676,566	T/C	0.11	0.81	-++--+-++-
rs1181884	3,676,597	T/C	0.88	0.81	+--++-+--+
rs12406474	3,676,771	T/C	0.75	0.83	+++--++---
rs1181883	3,677,932	T/C	0.61	0.66	+-+-+++--+
rs4648558	3,679,460	A/T	0.74	0.87	-++--++---
rs10910024	3,679,774	T/C	0.23	0.78	++-++--+++
rs1181875	3,681,830	T/C	0.89	0.14	---++---+-
rs1181874	3,682,069	A/C	0.57	0.44	++--+-+---
rs16824081	3,683,348	A/G	0.085	0.41	-+---+++-+
rs1181872	3,684,106	A/T	0.47	0.11	+++-+---+-
rs10910025	3,684,184	A/G	0.086	0.46	-+---+++-+
rs1181871	3,684,320	A/G	0.44	0.40	---+-+-+-+
rs1175551	3,688,644	T/C	0.44	0.41	---+-+-+-+
rs2275819	3,689,407	A/G	0.086	0.23	++---+++-+
rs1175550	3,691,527	A/G	0.77	0.16	+--+-+++-+
rs1175549	3,691,726	A/C	0.75	0.44	+--+-+-+-+
rs1891937	3,694,646	A/G	0.10	0.79	++----++-+
rs2887275	3,695,110	C/G	0.10	0.79	++----++-+
rs2799182	3,695,999	T/C	0.51	0.40	---+-+++-+
rs17411279	3,696,491	T/C	0.10	0.83	++----++-+
rs3737593	3,696,535	A/G	0.88	0.65	-+++++--+-
rs8379	3,696,889	A/C	0.51	0.40	---+-+++-+
rs17411356	3,697,034	A/G	0.90	0.83	--++++--+-
rs17411384	3,698,223	C/G	0.10	0.83	++----++-+
rs12563491	3,700,215	T/C	0.09	0.30	+----+++-+
rs2298228	3,700,849	C/G	0.98	0.66	-+++--+--+
rs2298227	3,701,662	T/C	0.88	0.64	-+++++--+-

Position = map position in base pairs; RAF = reference allele frequency; meta P = meta analysis P-value; Direction = effect direction.

* Reference (risk) allele listed first.

## Discussion

P73 belongs to the p53 family of cell survival regulators and presents several isoforms controlled by two main alternative promoters leading to transactivating (TAp73) and N-terminally truncated (DeltaNp73) isoforms [35]. TAp73 and DeltaNp73 were previously reported to exert pro- and anti-apoptotic effects, respectively, in different cellular systems including the central nervous system [32,36]. P73 is required for correct development of the cerebrum, with hydrocephalus and hippocampal dysgenesis occurring in complete p73 knockout mice [37,38]. Lack of P73 is inferred to result in a loss of the protective functions and this concept has been explored in the specific context of the neurodegenerative changes of AD [19,22]. Hence, in a series of biochemical and histological analyses we attempted to examine and extend these potentially important interactions between P73 and Aβ in the setting of a previously described mouse model [19]. Intercrosses of two genetic stocks with distinct neurological deficits is a common strategy in neurodegenerative disease research and can result in novel or enhanced phenotypic traits. In practice, though using the same APP transgene and the same *Trp73* null allele, we have not been able to demonstrate AD-like tau pathologies in compound mutant mice, nor in *Trp73* haploinsufficent mice of comparable ages to those described by the Wetzel *et al.* study, nor in mice homozygous null for DeltaNp73. We are now considering theoretical and practical parameters that may explain these discrepancies.

One potential divergence with the initial report [19] concerns the genetic backgrounds in the respective cohorts, which are enriched for the 129SvJae and C3H/C57BL6 strains for the Wetzel *et al.* and the present study, respectively, and which might affect the nature and tempo of tau pathology. However, there are three observations here that bear on this issue. First, the C3H/C57BL6 genetic background is able to support the formation of hyperphosphorylated tau in dystrophic neurites in TgCRND8 mice ([4] and Figure 2B). Second, we did not detect any tau pathology in the homozygous null DeltaNp73 model, which contains a contribution from the 129Sv background, being a mixture of 129SvJ and C57BL/6 backgrounds. Third, mice examined here over four times older than those reported by Wetzel *et al.* failed to demonstrate abnormal tau species (Figure 3B). Also, in a report by Cancino *et al.*[22], *Trp73*^+/-^ -dependent tau immunostaining was noted in two distinct genetic backgrounds, C129129SvJae and C57/BL6. Overall, these findings decrease the impetus to consider (hypothetical) strain background effects as a crucial determinant of the widely divergent results.

A second potential divergence between the two sets of studies is the euthanasia protocol: sodium pentobarbital overdose [19,22] versus cervical dislocation (used here). Previous studies have shown that phospho-specific tau antibodies (*e.g.* AT8, AT180, and CP13) are prone to detect signals in the axons of mice arising from anesthetic administration and tissue temperature differences [33]. This effect was observed in C57BL6 wild type male animals (*i.e.* harboring neither human tau, nor human APP transgenes). Our results obtained with adolescent C3H/C57BL6 wt mice extend previous observations by Planel *et al.* (Additional file 4: Figure S4) and demonstrate an effect of sodium pentobarbital administration on the generation of phospho-tau species detected by immunoblot. This effect was manifest in experiments with a time-span of minutes from the time of anaesthetic administration to the time of tissue harvest.

Cancino *et al.*[22] have disclosed that the use of frozen vs. immersion-fixed tissue as an important variable within their own dataset, with no tau immunostaining visible in paraffin-embedded tissue. This is an interesting effect but alone it cannot explain all divergent outcomes between two prior studies [19,22]. Thus, unlike Wetzel *et al*., we were unable to detect tau immunostaining in frozen sections from compound transgenic mice (Figure 2A), while signal was obtained in positive control (tau transgenic) animals. Also, this methodological issue has no bearing on our failure to detect insoluble forms of tau in biochemical analyses of frozen tissue samples (Figures 1 and 3). However, the data presented here and these recent studies by Cancino *et al.*[22] when taken together point to a gap in the earlier studies that may have affected the conclusions about P73’s role in AD pathogenesis [19].

Pathological forms of tau can be detected by histochemical and immunostaining in paraffin-embedded fixed tissue from human source material, as well as in a number of animal models of tauopathy [1,11,15,21,28]. Indeed, use of fixed tissue preparations in conjunction with silver staining procedures has been used to stage the progressive changes as AD advances in severity [1]. Tau is naturally phosphorylated and it is possible to hypothesize that labile over-phosphorylated forms of tau could exist early in a disease process (such that their detection is much dependent upon the applied protocol), but with these species then evolving to highly aggregated fibrillary material of the sort detected by standard histochemical stains. However, this type of graded scenario with qualitative and quantitative increments in severity is not supported by the current data on *Trp73* mice. Thus there is no published evidence from compound transgenic mice that suggest unusual tau species attributed to *Trp73* hemizygosity evolve quantitatively and/or qualitatively with time. Earlier reports [19,22] focus on compound transgenic mice within a 2-week timeframe in adolescent mice and, thus far, we have failed to find evidence of novel pathologies in compound transgenic mice that differ from the parental TgCRND8 mice (evaluations here at 45, 60, 70, 85 and 270 days of age; Figures 1 and 2, Additional file 1: Figure S1, Additional file 2: Figure S2). Based on these considerations we are led to consider whether immunostained structures described in 45-60 day-old mice [19] do not represent the end-product of misfolding and deposition *in situ* but might represent transient hyperphosphorylated forms of tau arising from an interaction between the P73 genotype and a systematic effect of the conditions used for tissue harvest and processing. This could explain the paradox as to how *Trp73*^+/-^ + TgCRND8 compound transgenic mice are reported to have immunostaining of structures derived from wt endogenous mouse tau at earlier ages than mice overexpressing mutant forms of human tau [11,16,21,28].

Orthogonal data to appraise a hypothetical intersection of P73 biology and AD pathogenesis can be sought from genetics. Previously the human *TP73* gene was described as having borderline significant association with AD in a small dataset and that decreased p73 expression occurs in some cases of AD [23]. However only tentative and un-replicated evidence for association with AD in a subset of cases negative for the APOE ε4 allele was reported (genotypic p-value = 0.02). Li *et al.* also reported an allelic expression bias for p73 only in a single control brain (not in AD samples) and that the allele weakly associated with AD did not change the promoter activity of *TP73*. Scacchi *et al.*[39] tested association of AD with four *TP73* SNPs, including two (rs227395 and rs1801173), which form a putative functional haplotype, another in the promoter region (rs3765728) and a synonymous coding SNP (rs1801174). Marginal evidence of association was obtained for rs3765728 (p=0.047) based on a comparison of the rare G/G homozygote with the other genotypes. There were no results for this SNP in our study because it is an unproven SNP according to build 131 of the HapMap database (http://www.ncbi.nlm.nih.gov) and thus could not be imputed. Our meta-analysis of 10 large AD GWAS datasets failed to show an association between AD and any variants in the *TP73* region. Furthermore, the *TP73* locus failed to show significant evidence for association in two other very large GWAS [40,41]. The lack of any evidence of association between *TP73* and AD in a large sample strongly suggests that variants in *TP73* are unlikely to be a critical determinant in the pathogenesis of AD. With regards to other varieties of genomic alteration, one concept proposed by Wetzel *et al.*[19] is that some individuals lose one copy of the genomic locus encompassing the *TP73* gene and, consequently, that haploinsufficiency for *TP73* may represent an AD susceptibility factor. This hypothesis was based in part on a previous study of CNVs [24]. However, in actuality, this report described un-validated CNVs in 95 individuals, the majority of whom were from a cancer-screening program. Our data, derived from an AD case-control sample comprising more than one thousand individuals, failed to identify any CNVs in *TP73*.

## Conclusions

Our investigation argues strongly against p73 as a locus conferring genetic risk for AD pathology in mouse models or in humans. While there is some evidence showing that p73 haploinsufficiency may cause neurodegeneration in mouse models by modulation of tau kinases, we found no evidence for robust tau pathologies in tissue sections and in biochemical fractionations of cell lysates from *Trp*73 haploinsufficient mice that deposit human Aβ peptide, nor in aged deltaNp73 homozygous null mice, nor any evidence that variants in the human *TP73* gene influence AD susceptibility. Overall, given the lack of evidence for evolving pathology in *Trp73*^+/-^ + TgCRND8 mice [19,22], or discernible pathology (as here), and the failure of genetic analyses to confirm an influence of *Trp73* in human cohorts, the concept of a significant role for P73 in AD pathogenesis is not supported.

## Materials and methods

### Transgenic mouse studies

#### Mouse stocks and genotyping

Analysis of the Trp73 haploinsufficient mice and of the DeltaNp73 mutant mice were performed at the University of Alberta, Edmonton, Canada and at the Université Catholique de Louvain, Brussels, Belgium, respectively. The *Trp73* haploinsufficient mice described in the studies of Wetzel *et al.*[19] were obtained from the same originator, Frank McKeon at Harvard University. The mice were genotyped in a PCR involving three primers: wt locus, 5’ GGG CCA TGC CTG TCT ACA AGA A 3’; common wt region unaffected by gene targeting, 5’ CCT TCT ACA CGG ATG AGG TG 3’; NEO gene, 5’ GAA AGC GAA GGA GCA AAG CTG 3’ generating PCR products of 600 bp and 400 bp for the wild type locus and the interrupted allele respectively. PCR conditions were 94°C 45 seconds, 64°C 45 seconds, 72°C 90 seconds for 35 cycles with Platinum Taq polymerase (Invitrogen). These mice (on the sixth backcross to C57BL6) were intercrossed with the same TgCRND8 mice that were also used by Wetzel *et al*. TgCRND8 mice [4] were maintained on an outbred C3H x C57BL6 background. Offspring were genotyped for the APP transgene array by a PCR involving primers 5' TGTCCAAGATGCAGCAGAACGGCTACGAAAA 3' lying in the human APP coding region and 5’ AGAAATGAAGAAACGCCAAGCGCCGTGACT 3’ lying within the 3’ untranslated region of the mRNA encoded by the Syrian hamster PrP cosmid cos.Tet. transgene vector [42]. PCR conditions were 94°C 30 seconds, 56°C 30 seconds 68°C 5 minutes for 35 cycles with Taq polymerase (Invitrogen) (Additional file 1: Figure S1). As an internal control for immunodetection procedures (below) we employed the TgTau(P301L)23027 [28] or the Tg30 [29] lines of transgenic mice (in Edmonton and Brussels, respectively) both expressing a 4-repeat spliced form of human tau cDNA and developing neurofibrillary tangles. These mice were genotyped with the following primer pair: Primer 1572: 5' -TGG ATC TTA GCA ACG TCC AGT CC 3' and Primer 1587: 5' CTC TCC TCT CCA CAA TTA TTG ACC G 3' representing exonic sequences that are not contiguous in the mouse chromosomal tau locus but lie juxtaposed within the tau cDNA insert in the cos.Tet transgene vector. DeltaNp73 mutant mice were described previously [32] and maintained in a 129SvJ, C57BL/6 Background according to European guidelines. All animal procedures were approved by the animal ethics committees of the University of Alberta and at the Université catholique de Louvain.

#### Western blot analysis

Western blot analysis of tau species in tissue lysates and detergent insoluble extracts were performed as described previously [28]. 6E10 antibody (Sigma) was used for immunodetection of APP by western blot (1:1000 dilution). t-tau antibody (Chemicon) was used at 1:1000 dilution for western blot analyses of all tau species. All other tau antibodies were used at 1:500 for western blots, with the exception of AT180 (epitope: phosphothreonine 231 and phosphoserine 235, 1:1000, Innogenetics). Other antibody sources were AT180 and AT8 (phosphoserine 202 and phosphothreonine 205, Thermo Scientific). CP13 (phosphoserine 202), PHF-1 (phosphoserine 396/404) and MC1 (a conformational-specific antibody recognizing amino acid residues 5-15 and 312-322) were a gift from Dr. Peter Davies.

#### Pathological analysis and immunohistochemistry

##### Trp*73 haploinsufficient mice*

Mice were maintained in accordance with Canadian Council for Animal Care (CCAC) guidelines. Generally, brains were removed after cerebral dislocation or anesthetic overdose (sodium pentobarbital, 140 mg/kg) and bisected in the mid-saggital plane. Neutral-buffered formalin-fixed tissue was batch processed on an automatic tissue processor overnight with vacuum on each station to aid penetration. Paraffin sections were cut at 5 microns and affixed to Fisherbrand Superfrost/Plus slides. Sections for immunohistochemistry were blocked in Dako Dual Endogenous Enzyme-Blocking Reagent and normal goat serum prior to incubation with primary antibody overnight at 4°C and processing via the Dako EnVision+ Dual Link System-HRP kit with end products visualized with DAB. For AT180 antibody, sections were processed through an epitope retrieval step of immersion in 10 mM citrate buffer pH6.0, 0.05% Tween 20, and heating in a pressure cooker for 2 minutes. Sections were lightly counterstained with hematoxylin, resin mounted and images were acquired on a Nikon 90*i* microscope. For processing by fluorescent microscopy mice were perfused with PBS and fixed with 4% paraformaldehyde. Brains were dissected, immersed overnight in fixative, rinsed with PBS and transferred to 30% sucrose for 24 hours. Cryosections were stored in an antifreeze solution (PBS:Glycerol:Ethylene Glycerol=2:1.5:1.5) before immunostaining. Sections were washed with PBS, exposed to Dako Dual Endogenous Enzyme-Blocking Reagent for 15 minutes, rinsed with 1x PBS, blocked with 2% normal goat serum for 1 hour and then incubated overnight with primary antibody (AT8, 1:50 or 1:100, as noted; CP 13, 1:50; MC1, 1:50; PHF1, 1:50; 6E10, 1:500) in antibody diluting solution. Subsequent to PBS washes sections were incubated with secondary antibody (Alexa Fluor® 594 goat anti-mouse IgG (H+L) at 0.01 mg/mL) for 2 hours at room temperature, PBS-washed and mounted.

##### DeltaNp73 mutant mice

Brains were removed after transcardial perfusion of 4% paraformaldehyde in PBS and were embedded in paraffin. 8 μm thick coronal sections, prepared at various rostrocaudal levels, were stained by immunohistochemistry with antibodies directed against specific phosphotau epitopes (PHF-1 antibodies); the signal was detected using a standard ABC kit. Sections were also stained with the Gallyas silver staining method.

### Human studies

Diagnostic, demographic and SNP data were obtained from 10 genome-wide association study (GWAS) datasets comprising a total of 8309 AD cases and 7366 controls that constituted the discovery sample in a previously published genome-wide association study (GWAS) of AD by the Alzheimer Disease Genetics Consortium (ADGC) [34]. The number of subjects in each dataset included in the association analysis of *TP73* SNPs is given in Table 1. Appropriate local Institutional Review Board approvals were obtained for all datasets. In addition, 1,093 Hispanic elderly [549 patients with late-onset AD (LOAD) and 544 controls] included in a GWAS conducted at Columbia University (CU) was used to investigate copy number variants (CNVs). Subjects in that study were self-reported Hispanics and unrelated to other participants, and underwent an in-person baseline interview on general health and functional ability using a standardized assessment, including medical history, physical and neurological examination and a neuropsychological battery. Each participant was examined at 18 to 24 month intervals thereafter with repeated interviews and examinations standardized to preserve consistency. This dataset was not included in the meta analysis of *TP73* SNPs because the genetic make-up of these individuals is distinct from European Caucasians and we were unable to identify a suitable reference population with haplotype data for genotype imputation. Appropriate local Institutional Review Board approvals were obtained for all datasets.

#### Copy Number Variant (CNV) analysis

The search for CNVs affecting the *TP73* locus was done in the CU dataset from array intensity files as previously described [43]. Briefly, the CNV analysis was performed using at least two software packages (iPattern and either PennCNV or QuantiSNP). CNVs identified by two independent algorithms have a >95% chance of being true positives and could be prioritized for follow-up validation.

#### Statistical analysis

*TP73* encompasses an approximately 80 kb region on chromosome 1. For the purpose of genetic association analysis, we evaluated all SNPs located in the transcribed region and within 2000 base pairs of the start and end sites of the gene. A total of 91 genotyped and well-imputed (r^2^>0.3) SNPs with minor allele frequencies > 0.01 in the *TP73* gene were identified, but only 54 of these were included in the analysis because marker information was not available for the other SNPs in all 10 datasets. Association of *TP73* SNPs with AD was evaluated by comparison of allele frequencies and with an additive model using logistic regression. We tested an additive model using a logistic regression framework developed in R to compute the strength of the association of disease status with dosage of the risk allele. Familial data in the MIRAGE study was analyzed separately and in two ways. The discordant siblings were analyzed using a family-based allelic association test, as implemented in FBAT [44]. SNP genotypes in the MIRAGE dataset were evaluated using generalized estimating equations (GEE) [45] in the R package [46]. While this population-based test is sensitive to population stratification, we found no evidence that population substructure was associated with disease in this sample (evaluated by regressing disease on the first 10 principal components generated by EIGENSTRAT [47]). We report the results of association from the GEE framework because the FBAT were limited by low power from uninformative, small nuclear families. The GEE results from the testing of the additive model were used in the meta-analysis for consistency across datasets. In order to obtain a robust estimate of association in the combined datasets, we conducted a meta-analysis of the p-values for the additive model from the 10 Caucasian datasets using the METAL software (http://www.sph.umich.edu/csg/abecasis/Metal/index.html). This software implements an inverse-variance meta-analysis to combine the association results across datasets. It sums the regression coefficients weighted by the inverse variance of the coefficients. The meta-analysis p-value of the association is then obtained from the summarized test statistic. The Versatile Gene-based Association Study (VEGAS) approach [48] was used to summarize the strength of association of *TP73* with AD based on the number of SNPs tested in the gene and size of the gene. This method computes a gene-based test statistic based on the SNP p-values within the gene, and then uses simulation to calculate an empirical gene-based p-value.

## Abbreviations

AD: Alzheimer’s disease; CNV: Copy Number Variant; NFT: Neurofibrillary tangles; SNP: Single nucleotide polymorphism.

## Competing interests

Authors declare no competing personal or financial interests.

## Authors’ contribution

BV contributed to the design of the human study, carried out the analyses summarized in Tables 1, 2 and S1 with ML; JL; RC, and drafted the manuscript; DV contributed to the design of the animal study, carried out the experiments illustrated in Figures 1, 2, 3, S1-S4 and drafted the manuscript; FT contributed to the design of the animal study involving the DeltaNp73 and carried out the experiments illustrated in Figure 4; ND contributed to the histochemical analysis illustrated in Figure 3D; JY contributed to the histochemical analyses of and carried out the experiment illustrated in Figures 2 and 3 and S3 with DV; KA; ER; J-PB contributed to the histochemical analysis illustrated in Figure 4A; MG; BS contributed to the histochemical analysis; CTB; SK participated in the design of the animal study; RM; PF; AMG contributed to the design of the animal study involving the DeltaNp73; PSGH; LAF; DW designed and coordinated the study and drafted the manuscript. All authors read and approved the final manuscript.

## Supplementary Material

Additional file 1: Figure S1 Longer exposure of western blot shown in the first column of Figure 1A from 45 days-old animals. See Figure 1. Again, “+/-“ in the case of *Trp73 *and APP indicates that these mice were heterozygous for null allele and hemizygous for the TgCRND8 APP transgene array, respectively. Click here for file

Additional file 2: Figure S2 Cerebral cortex in control and compound transgenic mice aged 70 days. The left-hand column shows a control TgTau^P301L ^mouse. No tau immunostaining is seen in 70 day-old compound mutant animals (2^nd ^column) with AT8, CP13, MC1 and AT180 antibodies. The cortex of this animal is however positive for Aβ-containing amyloid plaques (6E10 antibody, data not shown). Aged matched *Trp*73^+/- ^and TgCRND8 littermate controls also do not present any tau immunostaining, as illustrated in the 3^rd ^and 4^th ^columns, respectively. All panels, 10 x objective. Click here for file

Additional file 3: Figure S3 Biochemical analyses of soluble tau species in aged compound and *Trp73* hemizygous mice. Western blot analyses of phospho-specific tau species in soluble protein fractions obtained after fractionation of homogenates from sagittally-sectioned hemi-brains. Protein samples were derived from animals at 270 days of age. Tau antibodies, AT8, AT180, CP13 and PHF1 (rows 1-4, respectively), were used to evaluate pathological forms of tau in the samples while the APP-specific 6E10 antibody was used to confirm the presence of the APP transgene (row 5). A tau transgenic sample (left-hand lane) was included in the immunoblot series as a positive control (TgTau^P301L ^mouse, age 540 days). An actin immunoblot serves as loading control in the soluble fraction (row 6). Click here for file

Additional file 4: Figure S4 Western blot analyses of phospho-specific tau species in insoluble protein fractions obtained from anaesthetic treated or control C3HxC57 F1 mice. Western blot analysis was with CP13 and PHF1 antibodies. Genders are denoted by either “f” (female) or “m” (male). “Control” represents euthanasia by cervical dislocation, “Na-pentob” represents euthanasia by overdose with sodium pentobarbital. Loading controls were as per Figures 1 and 3. Click here for file

Additional file 5: Table S1 Association of *TP73 *SNPs with AD in the GWAS datasets. Click here for file
