# Supplementary figures and images for: Role of p73 in Alzheimer disease: lack of association in mouse models or in human cohorts
Source: Mol Neurodegener. 2013 Feb 15;8:10. doi: 10.1186/1750-1326-8-10 (PMC3614544; doi:10.1186/1750-1326-8-10)

## Supplemental Fig. 1

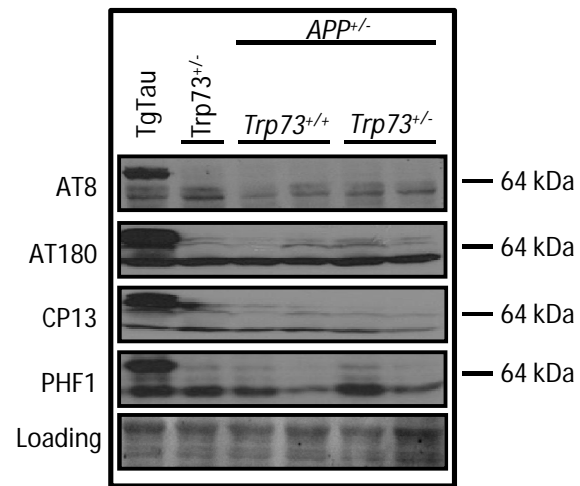

Supplement: Additional file 1: Figure S1 — Longer exposure of western blot shown in the first column of Figure 1A from 45 days-old animals. See Figure 1. Again, “+/-“ in the case of Trp73 and APP indicates that these mice were heterozygous for null allele and hemizygous for the TgCRND8 APP transgene array, respectively. [file 1750-1326-8-10-S1.pdf]

## Supplemental Fig. 2

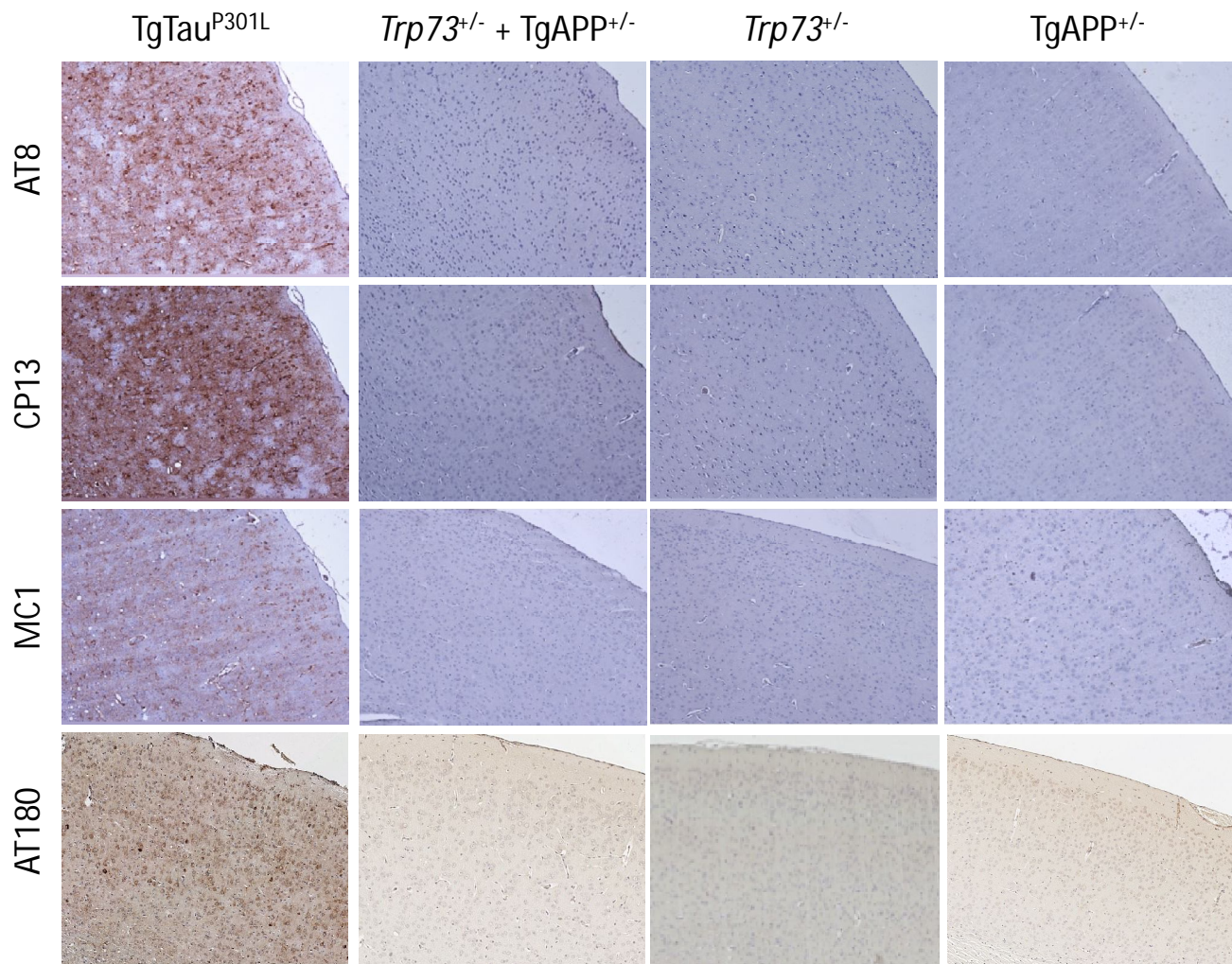

Supplement: Additional file 2: Figure S2 — Cerebral cortex in control and compound transgenic mice aged 70 days. The left-hand column shows a control TgTauP301L mouse. No tau immunostaining is seen in 70 day-old compound mutant animals (2nd column) with AT8, CP13, MC1 and AT180 antibodies. The cortex of this animal is however positive for Aβ-containing amyloid plaques (6E10 antibody, data not shown). Aged matched Trp73+/- and TgCRND8 littermate controls also do not present any tau immunostaining, as illustrated in the 3rd and 4th columns, respectively. All panels, 10 x objective. [file 1750-1326-8-10-S2.pdf]

## Supplemental Fig. 3

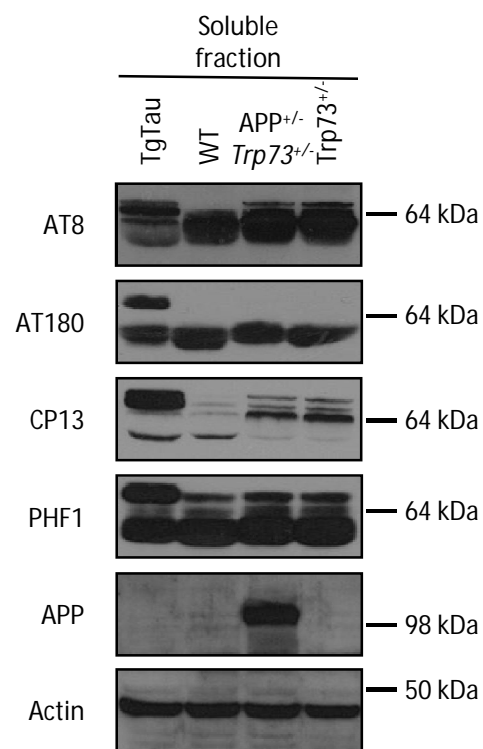

Supplement: Additional file 3: Figure S3 — Biochemical analyses of soluble tau species in aged compound and Trp73 hemizygous mice. Western blot analyses of phospho-specific tau species in soluble protein fractions obtained after fractionation of homogenates from sagittally-sectioned hemi-brains. Protein samples were derived from animals at 270 days of age. Tau antibodies, AT8, AT180, CP13 and PHF1 (rows 1-4, respectively), were used to evaluate pathological forms of tau in the samples while the APP-specific 6E10 antibody was used to confirm the presence of the APP transgene (row 5). A tau transgenic sample (left-hand lane) was included in the immunoblot series as a positive control (TgTauP301L mouse, age 540 days). An actin immunoblot serves as loading control in the soluble fraction (row 6). [file 1750-1326-8-10-S3.pdf]

## Supplemental Fig. 4

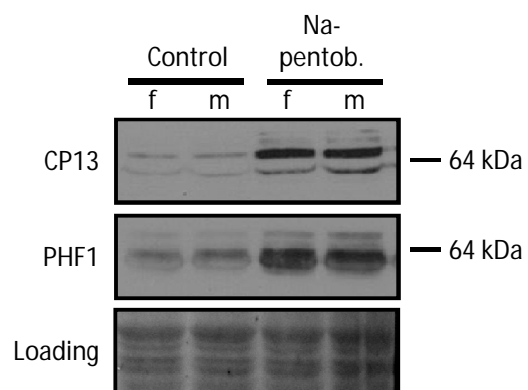

Supplement: Additional file 4: Figure S4 — Western blot analyses of phospho-specific tau species in insoluble protein fractions obtained from anaesthetic treated or control C3HxC57 F1 mice. Western blot analysis was with CP13 and PHF1 antibodies. Genders are denoted by either “f” (female) or “m” (male). “Control” represents euthanasia by cervical dislocation, “Na-pentob” represents euthanasia by overdose with sodium pentobarbital. Loading controls were as per Figures 1 and 3. [file 1750-1326-8-10-S4.pdf]
