# Supplementary material for: Role of p73 in Alzheimer disease: lack of association in mouse models or in human cohorts
Source: Mol Neurodegener. 2013 Feb 15;8:10. doi: 10.1186/1750-1326-8-10 (PMC3614544; doi:10.1186/1750-1326-8-10)
Supplement: Additional file 5: Table S1 — Association of TP73 SNPs with AD in the GWAS datasets. [file 1750-1326-8-10-S5.doc]

**Table S1:** Association of *TP73* SNPs with AD in the GWAS datasets.

|  |  |  |  |  |  | P-values for Individual Datasets (sample size) | | | | | | | | | |
| --- | --- | --- | --- | --- | --- | --- | --- | --- | --- | --- | --- | --- | --- | --- | --- |
|  |  |  |  |  |  | ACT | ADC1 | ADC2 | ADNI | GenADA | MIAMI | NIA-LOAD | OHSU | TGEN | MIRAGE |
| SNP | Position | Alleles * | RAF | Meta P | Direction | (2261) | (2081) | (898) | (441) | (1382) | (2231) | (3822) | (285) | (1357) | (1487) |
| rs2821021 | 3,524,142 | A/G | 0.70 | 0.71 | +--+-+-+-+ | 0.18 | 0.24 | 0.23 | 0.30 | 0.11 | 0.37 | 0.49 | 0.62 | 0.39 | 0.90 |
| rs4648538 | 3,543,492 | T/C | 0.27 | 0.56 | --+-+-+-++ | 0.98 | 0.74 | 0.74 | 0.37 | 0.20 | 0.55 | 0.79 | 0.17 | 0.98 | 0.12 |
| rs4276857 | 3,544,345 | T/C | 0.73 | 0.57 | ++-+-+-+-- | 0.98 | 0.74 | 0.74 | 0.37 | 0.20 | 0.53 | 0.79 | 0.17 | 0.98 | 0.12 |
| rs1128474 | 3,545,611 | G/A | 0.78 | 0.22 | -+-+---+-- | 0.87 | 0.43 | 0.67 | 0.76 | 0.27 | 0.87 | 0.66 | 0.29 | 0.31 | 0.036 |
| rs1885864 | 3,554,987 | T/C | 0.19 | 0.24 | +-+++++-++ | 0.99 | 0.20 | 0.35 | 0.94 | 0.28 | 0.87 | 0.59 | 0.48 | 0.24 | 0.029 |
| rs3818330 | 3,555,427 | T/G | 0.19 | 0.24 | +-+++++-++ | 0.99 | 0.20 | 0.35 | 0.94 | 0.28 | 0.87 | 0.60 | 0.48 | 0.23 | 0.029 |
| rs12027041 | 3,591,447 | C/G | 0.47 | 0.98 | +-++-----+ | 0.55 | 0.79 | 0.20 | 0.067 | 0.83 | 0.23 | 0.42 | 0.64 | 0.070 | 0.53 |
| rs3765770 | 3,635,980 | A/G | 0.036 | 0.76 | -+++--++-- | 0.61 | 0.45 | 0.94 | 0.75 | 0.54 | 0.20 | 0.86 | 0.44 | 0.55 | 0.35 |
| rs747828 | 3,636,225 | T/C | 0.96 | 0.78 | +---++--++ | 0.61 | 0.45 | 0.94 | 0.75 | 0.54 | 0.21 | 0.86 | 0.44 | 0.55 | 0.35 |
| rs747827 | 3,636,346 | A/C | 0.96 | 0.79 | +---++--++ | 0.61 | 0.45 | 0.94 | 0.75 | 0.54 | 0.22 | 0.86 | 0.44 | 0.55 | 0.34 |
| rs2236365 | 3,644,857 | C/G | 0.056 | 0.77 | --++---++- | 0.05 | 0.81 | 0.46 | 0.82 | 0.90 | 0.62 | 0.028 | 0.24 | 0.12 | 0.32 |
| rs6664760 | 3,648,267 | T/C | 0.96 | 0.79 | +---++--++ | 0.48 | 0.74 | 0.61 | 0.69 | 0.55 | 0.35 | 0.56 | 0.25 | 0.40 | 0.36 |
| rs6695978 | 3,648,344 | A/G | 0.041 | 0.80 | -+++--++-- | 0.48 | 0.75 | 0.61 | 0.69 | 0.54 | 0.36 | 0.56 | 0.25 | 0.41 | 0.35 |
| rs9662633 | 3,649,561 | A/G | 0.057 | 0.64 | -+++--++-- | 0.55 | 0.78 | 0.98 | 0.91 | 0.64 | 0.40 | 0.90 | 0.26 | 0.36 | 0.26 |
| rs12562437 | 3,651,030 | T/C | 0.041 | 0.80 | -+++--++-- | 0.46 | 0.75 | 0.60 | 0.69 | 0.55 | 0.38 | 0.56 | 0.25 | 0.41 | 0.35 |
| rs10910018 | 3,651,408 | A/G | 0.042 | 0.83 | -+++-+-+-- | 0.45 | 0.78 | 0.65 | 0.56 | 0.81 | 1.00 | 0.69 | 0.30 | 0.47 | 0.93 |
| rs12117836 | 3,657,758 | A/G | 0.37 | 0.91 | --+++--++- | 0.25 | 0.72 | 0.83 | 0.062 | 0.97 | 0.21 | 0.016 | 0.071 | 0.87 | 0.16 |
| rs2298222 | 3,659,656 | A/G | 0.30 | 0.80 | ---++--+++ | 0.77 | 0.77 | 0.79 | 0.063 | 0.99 | 0.34 | 0.16 | 0.19 | 0.65 | 0.33 |
| rs3737589 | 3,662,844 | A/G | 0.63 | 0.88 | ++---++--+ | 0.26 | 0.72 | 0.84 | 0.059 | 0.96 | 0.22 | 0.015 | 0.067 | 0.87 | 0.15 |
| rs12120656 | 3,665,866 | T/G | 0.37 | 0.89 | --+++--++- | 0.26 | 0.73 | 0.83 | 0.059 | 0.96 | 0.20 | 0.015 | 0.067 | 0.88 | 0.16 |
| rs4648554 | 3,668,003 | T/C | 0.63 | 0.91 | ++---++--+ | 0.26 | 0.74 | 0.83 | 0.059 | 0.97 | 0.18 | 0.015 | 0.068 | 0.84 | 0.16 |
| rs12128253 | 3,668,752 | A/C | 0.75 | 0.77 | -++--++--- | 0.95 | 0.66 | 0.62 | 0.11 | 0.85 | 0.20 | 0.18 | 0.10 | 0.43 | 0.30 |
| rs10797410 | 3,669,200 | A/G | 0.63 | 0.92 | ++---++--+ | 0.26 | 0.74 | 0.82 | 0.059 | 0.96 | 0.19 | 0.02 | 0.068 | 0.78 | 0.16 |
| rs10910022 | 3,669,499 | A/G | 0.97 | 0.74 | +---++--++ | 0.33 | 0.77 | 0.36 | 0.40 | 0.87 | 0.50 | 0.60 | 0.25 | 0.34 | 0.96 |
| rs1181889 | 3,671,026 | T/C | 0.88 | 0.95 | +--++-+--+ | 0.63 | 0.33 | 0.51 | 0.19 | 0.46 | 0.48 | 0.10 | 0.24 | 0.024 | 0.54 |
| rs1181888 | 3,671,790 | A/G | 0.88 | 0.93 | +--++-+--+ | 0.63 | 0.33 | 0.51 | 0.19 | 0.46 | 0.48 | 0.10 | 0.24 | 0.025 | 0.55 |
| rs12128669 | 3,676,076 | T/C | 0.25 | 0.78 | ---++--+++ | 0.99 | 0.77 | 0.70 | 0.11 | 0.83 | 0.19 | 0.22 | 0.10 | 0.43 | 0.33 |
| rs1181885 | 3,676,566 | T/C | 0.11 | 0.81 | -++--+-++- | 0.52 | 0.36 | 0.63 | 0.15 | 0.35 | 0.26 | 0.063 | 0.30 | 0.018 | 0.56 |
| rs1181884 | 3,676,597 | T/C | 0.88 | 0.81 | +--++-+--+ | 0.67 | 0.36 | 0.63 | 0.19 | 0.51 | 0.33 | 0.058 | 0.29 | 0.033 | 0.37 |
| rs12406474 | 3,676,771 | T/C | 0.75 | 0.83 | +++--++--- | 0.99 | 0.71 | 0.68 | 0.10 | 0.83 | 0.20 | 0.23 | 0.11 | 0.46 | 0.31 |
| rs1181883 | 3,677,932 | T/C | 0.61 | 0.66 | +-+-+++--+ | 0.81 | 0.77 | 0.84 | 0.47 | 0.73 | 0.91 | 0.024 | 0.020 | 0.36 | 0.11 |
| rs4648558 | 3,679,460 | A/T | 0.74 | 0.87 | -++--++--- | 0.97 | 0.75 | 0.55 | 0.08 | 0.91 | 0.45 | 0.43 | 0.055 | 0.49 | 0.22 |
| rs10910024 | 3,679,774 | T/C | 0.23 | 0.78 | ++-++--+++ | 0.97 | 0.79 | 0.47 | 0.09 | 0.82 | 0.25 | 0.26 | 0.11 | 0.55 | 0.14 |
| rs1181875 | 3,681,830 | T/C | 0.89 | 0.14 | ---++---+- | 0.05 | 0.87 | 0.43 | 0.98 | 0.31 | 0.97 | 0.98 | 0.46 | 0.55 | 0.047 |
| rs1181874 | 3,682,069 | A/C | 0.57 | 0.44 | ++--+-+--- | 0.16 | 0.57 | 0.84 | 0.0092 | 0.23 | 0.80 | 0.077 | 0.62 | 0.25 | 0.71 |
| rs16824081 | 3,683,348 | A/G | 0.085 | 0.41 | -+---+++-+ | 0.41 | 0.34 | 0.05 | 0.014 | 0.67 | 0.53 | 0.39 | 0.36 | 0.008 | 0.16 |
| rs1181872 | 3,684,106 | A/T | 0.47 | 0.11 | +++-+---+- | 0.92 | 0.48 | 0.75 | 0.0010 | 0.08 | 0.61 | 0.065 | 0.62 | 0.10 | 0.44 |
| rs10910025 | 3,684,184 | A/G | 0.086 | 0.46 | -+---+++-+ | 0.40 | 0.46 | 0.058 | 0.017 | 0.67 | 0.54 | 0.41 | 0.37 | 0.010 | 0.17 |
| rs1181871 | 3,684,320 | A/G | 0.44 | 0.40 | ---+-+-+-+ | 0.21 | 0.46 | 0.82 | 0.0017 | 0.22 | 0.66 | 0.069 | 0.87 | 0.20 | 0.82 |
| rs1175551 | 3,688,644 | T/C | 0.44 | 0.41 | ---+-+-+-+ | 0.22 | 0.50 | 0.83 | 0.00052 | 0.24 | 0.72 | 0.07 | 0.62 | 0.16 | 0.76 |
| rs2275819 | 3,689,407 | A/G | 0.086 | 0.23 | ++---+++-+ | 0.072 | 0.80 | 0.57 | 0.87 | 0.40 | 0.67 | 0.97 | 0.59 | 0.58 | 0.094 |
| rs1175550 | 3,691,527 | A/G | 0.77 | 0.16 | +--+-+++-+ | 0.93 | 0.52 | 0.41 | 0.42 | 0.27 | 0.21 | 0.17 | 0.56 | 0.035 | 0.67 |
| rs1175549 | 3,691,726 | A/C | 0.75 | 0.44 | +--+-+-+-+ | 0.80 | 0.20 | 0.29 | 0.52 | 0.19 | 0.20 | 0.10 | 0.15 | 0.13 | 0.92 |
| rs1891937 | 3,694,646 | A/G | 0.10 | 0.79 | ++----++-+ | 0.64 | 0.76 | 0.066 | 0.0046 | 0.52 | 0.53 | 0.24 | 0.55 | 0.11 | 0.29 |
| rs2887275 | 3,695,110 | C/G | 0.10 | 0.79 | ++----++-+ | 0.64 | 0.75 | 0.065 | 0.0045 | 0.51 | 0.51 | 0.24 | 0.55 | 0.11 | 0.29 |
| rs2799182 | 3,695,999 | T/C | 0.51 | 0.40 | ---+-+++-+ | 0.68 | 0.23 | 0.44 | 0.00088 | 0.08 | 0.39 | 0.063 | 0.11 | 0.18 | 0.92 |
| rs17411279 | 3,696,491 | T/C | 0.10 | 0.83 | ++----++-+ | 0.63 | 0.74 | 0.065 | 0.0044 | 0.48 | 0.49 | 0.24 | 0.56 | 0.12 | 0.30 |
| rs3737593 | 3,696,535 | A/G | 0.88 | 0.65 | -+++++--+- | 0.53 | 0.83 | 0.034 | 0.0042 | 1.00 | 0.81 | 0.12 | 0.66 | 0.23 | 0.41 |
| rs8379 | 3,696,889 | A/C | 0.51 | 0.40 | ---+-+++-+ | 0.67 | 0.23 | 0.44 | 0.0010 | 0.08 | 0.39 | 0.06 | 0.11 | 0.17 | 0.92 |
| rs17411356 | 3,697,034 | A/G | 0.90 | 0.83 | --++++--+- | 0.63 | 0.74 | 0.065 | 0.0044 | 0.48 | 0.49 | 0.23 | 0.55 | 0.13 | 0.30 |
| rs17411384 | 3,698,223 | C/G | 0.10 | 0.83 | ++----++-+ | 0.63 | 0.74 | 0.065 | 0.0047 | 0.48 | 0.49 | 0.23 | 0.55 | 0.13 | 0.30 |
| rs12563491 | 3,700,215 | T/C | 0.09 | 0.30 | +----+++-+ | 0.13 | 0.95 | 0.62 | 0.91 | 0.43 | 0.63 | 0.97 | 0.63 | 0.61 | 0.08 |
| rs2298228 | 3,700,849 | C/G | 0.98 | 0.66 | -+++--+--+ | 0.60 | 0.13 | 0.32 | 0.57 | 0.13 | 0.24 | 0.24 | 0.84 | 0.55 | 0.71 |
| rs2298227 | 3,701,662 | T/C | 0.88 | 0.64 | -+++++--+- | 0.52 | 0.82 | 0.035 | 0.0050 | 0.98 | 0.84 | 0.12 | 0.62 | 0.26 | 0.41 |

Position = map position in base pairs; RAF = reference allele frequency; meta P = meta analysis P-value; Direction = effect direction

* Reference (risk) allele listed first
